# 局限期小细胞肺癌治疗新格局：从传统放化疗到精准治疗的新进展

**DOI:** 10.3779/j.issn.1009-3419.2026.101.04

**Published:** 2026-02-20

**Authors:** Jingwen HAN, Keyao DAI, Zixi MENG, Jun HUANG

**Affiliations:** 510120 广州，广州医科大学附属第一医院胸外科，呼吸疾病国家重点实验室及国家呼吸系统疾病临床医学研究中心; Department of Thoracic Surgery, The First Affiliated Hospital of Guangzhou Medical University, State Key Laboratory of Respiratory Disease & National Clinical Research Center for Respiratory Disease, Guangzhou 510120, China

**Keywords:** 肺肿瘤, 放化疗, 免疫治疗, 靶向治疗, 分子分型, Lung neoplasms, Chemoradiotherapy, Immunotherapy, Targeted therapy, Molecular subtypes

## Abstract

局限期小细胞肺癌（limited-stage small cell lung cancer, LS-SCLC）是一种侵袭性强、预后差的恶性肿瘤。长期以来，依托泊苷联合铂类化疗联合同步胸腔放疗是其标准一线治疗方案，但多数患者仍面临复发与耐药。本文系统梳理近年来该领域的重大进展，旨在展现从传统放化疗向精准整合治疗转变的新格局。放疗技术持续优化，手术在严格筛选的早期患者中价值被重新确立；免疫治疗实现里程碑突破，ADRIATIC研究证实放化疗后Durvalumab巩固治疗可显著延长中位生存期（55.9 *vs* 33.4个月）；同时，基于分子分型的靶向治疗亦显示出潜力。本文阐明多模式治疗策略的优化方向，强调分子分型与生物标志物在个体化治疗中的指导意义，以期为临床实践提供参考，并为未来研究提供框架，从而整体提升LS-SCLC患者的生存预后。

## Abstract (en)

Limited-stage small cell lung cancer (LS-SCLC) is a highly aggressive malignancy with a poor prognosis. For a long time, the standard first-line treatment has been Etoposide plus Platinum-based chemotherapy combined with concurrent thoracic radiotherapy; however, most patients still face recurrence and drug resistance. This article systematically reviews major recent advances in the field, aiming to illustrate the new paradigm shifting from conventional chemoradiotherapy toward precision-integrated therapy. Radiotherapy techniques continue to be optimized, and the value of surgery has been reaffirmed in rigorously selected early-stage patients; immunotherapy has achieved a milestone breakthrough, with the ADRIATIC trial demonstrating that consolidation therapy with Durvalumab after chemoradiotherapy significantly extends median overall survival (55.9 *vs* 33.4 months); meanwhile, targeted therapies based on molecular subtyping also show promise. This paper clarifies the optimized directions of multimodal treatment strategies, emphasizes the guiding significance of molecular subtyping and biomarkers in individualized therapy, with the aim of providing reference for clinical practice, offering a framework for future research, and ultimately improving the survival outcomes of LS-SCLC patients as a whole.

肺癌是目前全球癌症发病和死亡的主要原因，2022年全球新增病例近250万例，死亡人数超过180万^[1]^。其中小细胞肺癌（small cell lung cancer, SCLC）在所有肺癌中占13%-15%^[2]^。烟草是SCLC发病最密切的危险因素，还有研究^[3,4]^表明，空气污染和氡与不吸烟者的SCLC之间可能存在联系。

1957年，退伍军人管理局肺部研究组根据肿瘤是否局限于单侧胸腔且能否被纳入一个安全的放射治疗野将SCLC分为局限期SCLC（limited-stage SCLC, LS-SCLC）和广泛期SCLC（extensive-stage SCLC, ES-SCLC），国际肺癌协会根据“肿瘤原发灶-淋巴结-转移（tumor-node-metastasis, TNM）”分期优化该标准，将LS-SCLC定义为除T3-T4外的任何 T、任何N和M0，只有出现远处转移（M1）才会被定义为ES-SCLC^[5]^。低剂量螺旋计算机断层扫描（low-dose computed tomography, LDCT）对整体肺癌的早期筛查起到至关重要的作用，但是由于CT筛查在SCLC的早期阶段检测能力非常有限，且SCLC早期症状隐匿、进展迅猛，LDCT筛查似乎并没有改善SCLC的生存结果^[6]^。

分子层面，*TP53*和*RB1*基因缺失导致G_1_/S检查点活性消失在SCLC患者中非常普遍，此外*CREBBP*、*PTEN*和*EP300*也是SCLC中常见的基因突变^[7]^。近年来，基于分子特征的研究将SCLC分为4种不同的亚型：SCLC-A、SCLC-N、SCLC-P和SCLC-Y，这些亚型在关键转录因子（*ASCL1*、*NEUROD1*、*POU2F3*和*YAP1*）的表达和免疫微环境上存在差异，为开发新的靶向和免疫治疗策略提供了潜在方向^[8]^。

SCLC的预后通常较非小细胞肺癌（non-small cell lung cancer, NSCLC）更差。美国监测、流行病学和最终结果（Surveillance, Epidemiology and End Results, SEER）数据^[9]^分析表明，SCLC的5年相对生存率仅有6.4%，1983-2012年的中位生存期仅为7个月。LS-SCLC的预后尽管优于ES-SCLC，但仍不乐观，过去数十年其治疗疗效进入了平台期。目前，LS-SCLC的标准一线治疗方案是依托泊苷联合铂类化疗并同步进行早期胸腔放疗。然而，尽管初始治疗有效率很高，但多数患者最终面临复发、转移及耐药。优化现有放化疗策略、整合免疫检查点抑制剂（immune checkpoint inhibitors, ICIs）、开发针对特定分子亚型的靶向药物，已成为改善LS-SCLC患者生存的核心方向。本综述系统回顾LS-SCLC标准治疗模式，讨论关键争议，并展望免疫治疗与分子分型指导下的精准治疗前景。

## 1 化疗

化疗是LS-SCLC治疗的基石，依托泊苷联合顺铂（Cisplatin-Etoposide, EP）方案自1980年代以来一直是标准一线治疗。然而，尽管初始反应良好，多数患者在治疗后数月内复发^[10]^。多年来，多项研究试图通过不同策略改善EP方案的疗效，在其基础上联合其他细胞毒药物（如紫杉醇或异环磷酰胺）、在同步放化疗（concurrent chemoradiotherapy, CCRT）前后更换依托泊苷，或采用不同的化疗组合方案。然而，这些尝试并未显著改善患者总生存期（overall survival, OS）^[11]^。一项III期随机试验^[12]^显示，大剂量表柔比星联合顺铂（Epirubicin plus Cisplatin, EPI-DDP）方案在客观缓解率（objective response rate, ORR）、无进展生存期（progression-free survival, PFS）和OS方面与EP方案均无显著差异，但EPI-DDP方案的3/4级中性粒细胞减少症发生率和治疗相关死亡率更低，提示其可能是一个毒性更低的替代选项。另一方面，日本JCOG0202研究^[13]^则聚焦于巩固治疗阶段，在LS-SCLC中，于初始EP方案联合CCRT后，采用伊立替康联合顺铂（Irinotecan plus Cisplatin, IP）方案进行巩固治疗，并未能改善OS（中位OS：2.8 *vs* 3.2年）。

综上，现有证据表明EP方案仍是LS-SCLC的疗效基准，EPI-DDP等低毒性方案未能超越其疗效，巩固阶段替换化疗药物亦未见生存获益。化疗研究的价值不在于替代EP，而在于探索其与放疗、免疫治疗的协同增效。

## 2 放疗

放疗在LS-SCLC治疗中具有重要地位。在化疗的基础上联合放疗，能够显著提高LS-SCLC患者的存活率，早期CCRT疗效优于序贯放化疗，其中位OS为27.2 *vs* 19.7个月^[14]^。LS-SCLC的放疗剂量和分割模式的选择也是临床研究的重点领域。目前常用的放疗模式为45 Gy/30次和60 Gy/40次，后者显著改善了LS-SCLC患者的OS，且未增加急性或长期毒性^[15]^。随着放疗技术的不断发展，调强放疗（intensity-modulated radiotherapy, IMRT）、质子束治疗（proton beam therapy, PBT）等精准放疗技术逐渐应用于LS-SCLC。IMRT通过为中心病灶区施以较高辐射剂量，同时向存在亚临床病灶或高风险区域给予较低剂量，可在提升OS获益的同时，显著降低辐射相关毒性反应的发生风险^[16]^。

PBT凭借其独特的物理特性可进一步降低心脏、肺等危及器官受照剂量。一项针对LS-SCLC的前瞻性研究^[17]^显示，与IMRT相比，PBT显著降低了脊髓、心脏和肺的平均剂量。该研究中位随访14个月，1及2年局部控制率分别为85%与69%，1及2年OS率分别为72%与58%，中位OS为28.2个月；≥3级食管炎、肺炎等发生率均为3.3%。这些初步数据表明PBT用于LS-SCLC CCRT具有不俗的疗效与较好的安全性，但其长期生存优势仍需大规模前瞻性研究进一步验证^[17-19]^。

立体定向放疗（stereotactic body radiation therapy, SBRT）可以在更少的疗程内提供集中的高强度辐射，从而降低各种放疗并发症发生率。对于临床分期为I期（cT1-2N0）但因合并症或肺功能差等原因无法耐受手术的SCLC患者，SBRT已成为一种重要的根治性局部治疗替代方案。一项长达25年的回顾性研究^[20]^证实，对于因高龄、肺功能差或合并症而无法手术的I期SCLC患者，SBRT安全性良好，无治疗相关死亡，且能显著延长OS，中位OS达21个月，5年OS率为30%。此外，接受SBRT后联合辅助化疗的患者显示出更优的生存趋势，强调了局部治疗后进行全身治疗的重要性。

预防性颅脑照射（prophylactic cranial irradiation, PCI）是SCLC治疗的另一重要组成部分，但具有一定的神经认知毒性^[21]^。为减轻这一不良反应，海马回避性PCI（hippocampal avoidance-PCI, HA-PCI）被提出并应用于临床。研究^[22]^表明，HA-PCI在有效保留患者认知功能的同时，其脑转移控制率和OS与常规PCI相比无显著差异。随着脑部磁共振成像（magnetic resonance imaging, MRI）成为常规分期手段，对于LS-SCLC患者是否应常规行PCI出现了争议。近期研究^[23-25]^提示，采用脑部MRI监测引导下的SBRT作为挽救治疗，其生存结局与接受PCI的患者相比并无显著差异。该结果主要归因于高分辨率MRI提升了微小脑转移灶的早期检出能力，为及时行SBRT干预创造条件；而SBRT对局灶病变控制率高且认知损伤小。因此，“MRI监测+SBRT”策略将PCI从“一刀切”的预防转向基于个体复发风险的精准干预，避免低风险患者承受不必要的神经毒性。

## 3 手术

手术在SCLC中的作用历来有限，因多数患者在诊断时已处于晚期。一项Cochrane系统综述^[26]^指出，在I-III期SCLC的综合治疗中，手术切除的价值非常有限。但受制于原始数据的时效性与证据质量，该结论参考价值有待商榷。随着筛查技术的进步和早期诊断率的提高，手术在高度选择的LS-SCLC患者中的价值逐渐受到重视。

多项研究为手术在特定分期患者中的价值提供了有力支持。一项针对134对配对患者的回顾性研究^[27]^表明，对于LS-SCLC，接受根治性肺切除术联合化疗的患者中位OS（22 *vs* 11个月）和5年OS率（27% *vs* 4%）均显著优于非手术治疗组，且局部复发率大幅降低（15% *vs* 55%）。近年的研究^[28]^进一步明确，对于I期SCLC患者，接受化疗联合手术的中位OS和5年OS率显著优于标准的化疗联合放疗（77 *vs* 28个月，54.3% *vs* 29.1%）。

对于LS-SCLC的手术方式选择，基于人群的研究^[29,30]^证据表明，肺叶切除术是首选且能带来最佳生存预后的术式。若患者无法耐受肺叶切除，则肺段切除术的预后优于楔形切除术，视为适用于特定患者的合理替代选择。

一项基于SEER数据库的倾向评分匹配研究^[28]^显示，手术联合化疗相比单纯手术能显著延长LS-SCLC患者的中位OS（35 *vs* 23个月）。这与欧洲肿瘤内科学会指南和美国国立综合癌症网络指南的推荐相吻合，对于经过严格筛选评估的临床I-II期（cT1-2N0）SCLC患者，以完全切除（R0）为目标的手术联合术后辅助治疗可作为多模式治疗的一部分^[31,32]^。当前证据进一步表明，对于经过选择的LS-SCLC患者，手术整合入多模式治疗的时序需依据精确的临床分期而定，两种策略均能带来生存获益，但确保完全切除（R0）和接受化疗是成功的关键^[33]^。此外，有研究^[34]^通过对205例手术切除的SCLC患者进行分析，发现II期患者的5年OS率与I期患者相当（65.5% *vs* 63.8%），提示手术适应证可能谨慎扩展至伴有肺门淋巴结转移（N1）的II期患者。

对于因高龄、肺功能差或合并症多而无法耐受手术的cT1-2N0患者，SBRT是一种安全有效的根治性替代方案。一项为期25年的回顾性研究^[20]^显示，术后病理淋巴结阴性患者中位OS为48个月，5年OS率约48%。对于因高龄、肺功能差或合并症多而无法耐受手术的I期SCLC患者，SBRT则成为一种安全有效的根治性替代方案，其中位OS可达21个月，5年OS率约30%。综上，手术是cT1-2N0患者获得最佳生存潜力的首选局部治疗方式，而对于不可手术者，SBRT提供了有效的根治性选择，二者均需整合至包含全身治疗的多模式治疗框架中。

近年来，基于分子特征的SCLC分型为理解手术患者的预后差异提供了新视角。一项针对386例手术切除标本的国际多中心研究^[35]^证实，神经内分泌亚型（SCLC-A, SCLC-N, SCLC-AN）与较差的OS显著关联，而非神经内分泌亚型（SCLC-P和SCLC-QN）则预后更佳。值得注意的是，高*ASCL1*表达是独立的负面预后因素，而高*POU2F3*表达则与生存改善有关。这一发现强调，对于接受手术的LS-SCLC患者，术后肿瘤组织的分子分型可能有助于更精准地评估复发风险并指导辅助治疗策略。

## 4 免疫治疗

虽然ICIs未获批准用于LS-SCLC，但ICIs在ES-SCLC的一线治疗中已确立了重要地位，尤其是程序性细胞死亡受体1（programmed cell death protein-1, PD-1）/程序性细胞死亡配体1（programmed cell death ligand 1, PD-L1）抑制剂（阿特珠单抗和度伐利尤单抗）为部分患者带来了可观的生存获益^[36]^。近期一项多中心真实世界研究^[37]^证实，与单纯EP化疗相比，一线联合PD-1/PD-L1抑制剂能显著延长患者的PFS，并在匹配后队列中显示出OS的显著获益（中位OS：29.5 *vs* 20.0个月），且未显著增加治疗相关不良事件。目前，多项临床研究正在评估阿特珠单抗、度伐利尤单抗、派姆单抗、纳武单抗和伊匹木单抗等ICIs与CCRT序贯或联合用于LS-SCLC一线治疗的疗效与安全性。

巩固治疗方面，ADRIATIC试验具有里程碑式的意义，证实了在同步放化疗后未进展的LS-SCLC患者接受度伐利尤单抗巩固治疗兼具良好的安全性及显著的生存获益^[38]^。该III期研究显示，与安慰剂相比，度伐利尤单抗可显著延长中位OS（55.9 *vs* 33.4个月）和中位PFS（16.6 *vs* 9.2个月），且安全性可控。免疫治疗重要研究的关键数据汇总于表1^[38-47]^。

**表1:** LS-SCLC免疫治疗的主要临床试验汇总

Trial	Primary completion year	Phase	Arms and Interventions	Primary outcome measures
ADRIATIC (NCT03703297)^[38]^	2024	Phase III	Durvalumab consolidation therapy vs placebo	Median OS: 55.9 vs 33.4 mon (HR=0.73, P=0.01); Median PFS: 16.6 vs 9.2 mon (HR=0.76, P=0.02)
KEYLYNK-013 (NCT04624204)^[39]^	2027 (estimated)	Phase III	Pembrolizumab+CCRT followed by Pembrolizumab±Olaparib vs placebo+CCRT followed by placebo	PFS and OS (results pending)
SINCE-01^[40]^	2023	Phase II	Sintilimab+CCRT	2-yr PFS rate: 59.1%; 2-yr OS rate: 69.3%
NRG-LU005 (NCT03811002)^[41]^	2024	Phase II/III	Atezolizumab+CCRT vs CCRT	3-yr survival rate: 44.7% vs 50.3%
AdvantTIG-204 (NCT04866017)^[42]^	2023	Phase II	Tislelizumab+Ociperlimab+CCRT vs CCRT	Median PFS: 13.2 vs 9.5 mon (HR=0.80, P=0.2414)
STIMULI (NCT02046733)^[43]^	2020	Phase II	Nivolumab+Ipilimumab consolidation therapy vs observation	Median OS: NR vs 32.1 mon (HR=0.95, P=0.82); Median PFS: 10.7 vs 4.5 mon (HR=1.02, P=0.93)
SHR-1316-III-302 (NCT04691063)^[44]^	2025	Phase III	Adebrelimab+CCRT	Median PFS: 17.9 mon; 2-year OS rate: 64.3%
NCT03585998^[45]^	2021	Phase II	Durvalumab+CCRT	Median PFS: 14.4 mon (95%CI: 10.3-not reached); 24-mon PFS rate: 42.0%; 24-mon OS rate: 67.8%
NCT04418648^[46]^	2024	Phase II	Toripalimab+CCRT vs observation	Median PFS: NR vs 12.3 mon (HR=0.47, P=0.04)
LungMate-005 (NCT04539977)^[47]^	2023	Phase II	Neoadjuvant TQB2450+chemotherapy followed by surgery/radiotherapy and TQB2450 maintenance	ORR: 92.5% (95%CI: 83.9%-100%); 24-mon OS rate in surgical group: 85.7% (95%CI: 72.0%-100%)

LS-SCLC: limited-stage small cell lung cancer; CCRT: concurrent chemoradiotherapy; CI: confidence interval; HR: hazard ratio; ORR: objective response rate; OS: overall survival; PFS: progression-free survival; NR: not reached.

然而，在肯定其突破的同时也需理性审视。ADRIATIC研究入选人群主要为放化疗后未进展、世界卫生组织（World Health Organization, WHO）体能状态评分0-1分的患者，其结论在更广泛人群中的普适性仍需验证。长期安全性数据尚在积累，免疫相关不良反应的风险需持续关注。此外，部分患者仍面临原发或继发耐药，未来需探索联合策略与预测标志物。

与此同时，其他免疫联合策略也在积极探索中。一项I/II期研究显示，帕博利珠单抗联合同步放化疗的中位PFS和OS分别为19.7和39.5个月^[48]^。基于此，III期KEYLYNK-013研究^[39]^正在进行中，旨在评估帕博利珠单抗联合CCRT后，序贯帕博利珠单抗联合或不联合聚腺苷二磷酸核糖聚合酶[poly (ADP-ribose) polymerase, PARP]抑制剂奥拉帕尼的疗效。该研究设计基于临床前证据，表明PARP抑制剂可增强PD-1阻断的抗肿瘤活性，并与放化疗产生协同作用。但并非所有联合策略均显示优势，NRG-LU005研究^[41]^发现，在CCRT中加入Atezolizumab并未显著改善OS。此外，联合治疗可能增加治疗相关性肺炎等不良事件的风险，对毒性管理提出了更高要求^[40]^。

总体而言，当前证据更支持巩固治疗作为LS-SCLC免疫治疗的标准策略，尤其是基于III期随机试验的阳性结果。联合治疗虽具潜力，但仍处于探索阶段，需更多III期研究验证其生存获益与安全性。

免疫治疗的整合应用已向前延伸至新辅助治疗阶段。一项针对6例III期LS-SCLC患者的回顾性研究^[49]^采用度伐利尤单抗联合EP方案进行新辅助治疗，所有患者均达到部分病理缓解，治疗安全性良好且未延误手术。Song等^[50]^的研究进一步比较了新辅助免疫化疗与单纯新辅助化疗的疗效，结果提示联合免疫治疗更有利于肿瘤降期、改善肺功能并促进病理学上的肿瘤消退，两者安全性相当。Zhu等^[51]^的研究也支持这一发现，术前免疫化疗在ORR、手术切除率及病理完全缓解率方面均显示出优势，且未显著增加高级别不良事件。近期一项II期研究^[47]^证实，新辅助免疫化疗后手术患者的病理完全缓解率达42.9%，且24个月OS率达85.7%。上述结果表明，新辅助免疫化疗可为初始不可切除或高风险患者创造手术机会并改善长期预后，但其生存获益仍需大规模随机对照试验验证。

为了筛选出最可能从免疫治疗中获益的人群，研究者们正积极寻找有效的预测性生物标志物。不同于NSCLC，SCLC的PD-L1表达水平通常较低且异质性强，其预测价值存在争议^[52]^。而高肿瘤突变负荷虽在ES-SCLC中显示出与ICIs疗效的相关性，但在LS-SCLC中的预测作用仍需进一步验证^[53,54]^。其他潜在的生物标志物，如肿瘤浸润淋巴细胞的组成、免疫微环境分型等，也处于探索阶段^[55]^。

在此背景下，循环肿瘤DNA（circulating tumor DNA, ctDNA）的动态监测作为一种无创液体活检技术，为实时评估肿瘤负荷和预测免疫治疗疗效提供了新视角。一项2025年的前瞻性研究^[56]^显示，诱导化疗后、放疗前的ctDNA状态结合影像学肿瘤缩小率，可构建一套风险分层模型，精准筛选需强化治疗的高危人群，并为低危患者避免过度治疗提供依据。

目前基于关键转录因子表达谱的SCLC分子分型为理解肿瘤生物学异质性提供了新视角。近期有研究^[57-59]^表明，在4种亚型中，SCLC-I亚型可能对免疫治疗更具敏感性，且往往具有更优的生存结局。具体而言，SCLC-Ia亚型对化疗联合免疫治疗应答良好；SCLC-Ib亚型则因兼具免疫抑制特征与高基因组不稳定性，对免疫治疗的反应可能进一步增加。这些发现为SCLC的免疫个体化治疗提供了新方向。

## 5 靶向治疗

与驱动基因突变丰富的NSCLC不同，SCLC因普遍存在*TP53*与*RB1*功能缺失且缺乏高频可成药驱动突变，长期缺乏有效的靶向治疗药物^[60]^。这一困境在LS-SCLC中尤为突出，尽管以根治为目标，但同步放化疗后多数患者仍面临复发，长期生存不理想。因此，靶向治疗的探索核心在于将ES-SCLC或复发场景中具潜力的药物有效整合至根治性治疗框架，或作为维持治疗以延缓复发、改善生存。

近年来，针对特定分子亚型的靶向治疗取得了突破性进展，为LS-SCLC提供了新的干预思路。Delta样配体3（delta-like ligand 3, *DLL3*）在超过80%的SCLC肿瘤细胞表面高表达，尤其在神经内分泌特征显著的SCLC-A亚型中表达最高，而在正常组织中几乎不表达，使其成为理想的治疗靶点^[61]^。Rovalpituzumab tesirine（Rova-T）作为首个靶向*DLL3*的抗体药物偶联物（antibody drug conjugate, ADC），在早期研究^[62]^中显示出潜力。然而，在TAHOE和MERU两项III 期随机对照试验^[63,64]^中均未能证实其优于现有标准治疗，且毒性较大。但是其失败为后续*DLL3*靶向疗法的开发提供了重要经验。

当前，*DLL3*靶向治疗LS-SCLC的重心已转向双特异性抗体Tarlatamab。Tarlatamab可同时结合肿瘤细胞表面的*DLL3*与T细胞表面的CD3^[62]^，在复发/难治性ES-SCLC的关键III期试验（DeLLphi-304）中，相较于标准化疗，显著延长了患者的中位OS（13.6 *vs* 8.3个月）^[65]^。基于此，针对LS-SCLC的III期研究DeLLphi-306正在评估Tarlatamab作为CCRT后巩固维持治疗的疗效与安全性，标志着靶向治疗正向LS-SCLC的根治后阶段前移^[66]^。目前，Tarlatamab在中国尚未获批上市，但已获准开展临床试验，并被纳入多项针对SCLC的国内临床研究计划。后续相关研究应特别关注患者肿瘤的分子亚型，尤其是*DLL3*高表达的SCLC-A亚型人群，旨在实现“精准巩固”。

除了*DLL3*，*B7-H3*（*CD276*）作为另一个在SCLC中普遍表达且与免疫逃逸和不良预后有关的靶点，也备受关注^[67]^。其中，由中国药企自主研发的*B7-H3*靶向ADC药物HS-20093临床进展迅速，其I期研究^[68]^在复发性SCLC患者中的ORR达44.2%，中位PFS和OS分别为5.7和14.6个月，目前已在国内外进入II期临床试验阶段。此外，靶向B7-H3的抗体偶联药物Ifinatamab deruxtecan（I-DXd）同样显示出潜力，II期研究iDeate-Lung01显示其在经治ES-SCLC患者中的ORR为54.8%，中位OS为11.8个月，对基线脑转移患者亦表现出良好的颅内应答；全球III期研究iDeate-Lung02正在进一步评估其疗效与安全性^[69]^。虽然当前数据主要来自ES-SCLC，但其作用机制和初步疗效为在LS-SCLC中的应用提供了理论依据。I-DXd目前尚未在中国获批上市，但已在国内启动针对SCLC等实体瘤的临床研究，未来有望为国内复发SCLC患者提供新的治疗选择。此外，其他新兴靶点如*Trop-2*、*SEZ6*等及其相应药物也处于探索中，进一步丰富了LS-SCLC的潜在靶向治疗选择。

靶向治疗在LS-SCLC中的成功应用，关键在于精准整合策略与患者筛选。一方面需将新型药物有效融入多模式治疗体系，明确其与放化疗、免疫治疗的最佳组合时序；另一方面需借助生物标志物进行分层筛选，结合不同分子亚型的治疗敏感性，为优化联合策略提供依据。

## 6 小结

LS-SCLC的治疗正超越以放化疗为核心的传统模式，迎来多维度突破。免疫治疗的突破，尤其是CCRT后Durvalumab巩固治疗，显著改善了患者生存，而靶向药物如Tarlatamab为未来治疗提供了新方向。此外，手术在严格筛选的早期患者中重新确立其治疗价值，而SBRT则为不可手术的LS-SCLC患者提供了有效的根治性替代选择。

未来LS-SCLC治疗的发展方向将更加强调精准整合与个体化分层。一方面，通过分子分型及生物标志物识别不同亚群对不同治疗方式的敏感性差异，实现治疗策略的精细化匹配；另一方面，不断优化治疗时序与组合，全面提升LS-SCLC患者的长期生存结局。
